# Lactic acid bacteria and endogenous ethanol mediate proton pump inhibitor-associated MASLD: a multicohort cross-sectional mediation analysis

**DOI:** 10.1080/19490976.2026.2664712

**Published:** 2026-05-03

**Authors:** Mark Davids, Hilde Herrema, Albert K. Groen, Henrike Galenkamp, Aeilko Zwinderman, Joonatan Palmu, Aki Havulinna, Teemu Niiranen, Rob Knight, Yaïr Acherman, Rutger Franken, Joanne Verheij, Michael Dukas, Dutch Liver Pathology Panel, Jasmohan Bajaj, Cristina Llorente, Bernd Schnabl, Max Nieuwdorp, Abraham Meijnikman

**Affiliations:** a Department of Experimental Vascular Medicine, Amsterdam UMC, University of Amsterdam, Amsterdam, The Netherlands; b Department of Public and Occupational Health, Amsterdam Public Health (APH), Amsterdam UMC, University of Amsterdam, Amsterdam, The Netherlands; c Department of Clinical Epidemiology, Biostatistics and Bioinformatics, Amsterdam UMC, University of Amsterdam, Amsterdam, The Netherlands; d Division of Medicine, Turku University Hospital and University of Turku, Turku, Finland; e Department of Public Health and Welfare, Finnish Institute for Health and Welfare, Helsinki, Finland; f Department of Computing, Turku University Hospital and University of Turku, Turku, Finland; g Department of Pediatrics, Department of Computer Science & Engineering, Shu Chien-Gene Lay Department of Bioengineering, and Halıcıoğlu Data Science Institute, University of California, San Diego, USA; h Department of Metabolic and Bariatric Surgery, Spaarne Gasthuis, Hoofddorp, The Netherlands; i Department of Pathology, UMC, University of Amsterdam, Cancer Center Amsterdam, Amsterdam, The Netherlands; j Department of Pathology, Erasmus Medical Center Cancer Institute, Erasmus University Medical Center, Rotterdam, The Netherlands; k Division of Gastroenterology, Hepatology, and Nutrition, Virginia Commonwealth University and Richmond VA Medical Center, Richmond, VA, USA; l Department of Medicine, University of California San Diego, La Jolla, CA, USA; m Department of Internal Medicine, section Vascular Medicine, Amsterdam UMC, University of Amsterdam, Amsterdam Cardiovascular Sciences, Amsterdam, The Netherlands; n Tytgat Institute for Liver and Intestinal Research, Amsterdam Gastroenterology Endocrinology Metabolism, Amsterdam University Medical Center, Location AMC, University of Amsterdam, Amsterdam, The Netherlands

**Keywords:** MASLD, gastrointestinal microbiome, proton pump inhibitors, mediation analysis, fermentation, lactobacillales, ethanol, small intestine, obesity

## Abstract

**Background & aims:**

Proton pump inhibitor (PPI) use has been associated with metabolic dysfunction associated with steatotic liver disease (MASLD) in multiple studies. While the association is confounded by various risk factors, such as BMI and age, a potential mediating factor of the microbiome has been suggested. In this study, we aimed to identify bacterial clades with the highest mediating potential and evaluate the serially mediated path through microbially derived endogenous ethanol.

**Methods:**

Microbiome mediation analysis of PPI use and MASLD was conducted in two cohorts. In a bariatric surgery cohort (*n* = 122), liver biopsy-proven steatosis grade and postprandial ethanol concentrations were used as outcomes. In the HELIUS cohort (*n* = 2440), a general population cohort study, mediation was performed using the Fatty Liver Index (FLI) score. The strongest associations were validated in the FINRISK cohort (*n* = 7066).

**Results:**

Several bacterial taxa, which are predominantly found in the small intestine, showed a potential role in mediating the effects of PPIs on MASLD, postprandial ethanol levels, and FLI score. The Lactobacillales order showed the strongest mediating potential across the outcomes tested in both discovery cohorts. A notable serial mediation pathway was identified, linking PPI use to MASLD via Lactobacillales abundance and postprandial plasma ethanol concentrations. The mediating role of Lactobacillales in the association between PPI use and FLI scores was confirmed in the final study cohort.

**Conclusions:**

Data from multiple cross-sectional cohort studies support a mediating potential of the microbiome in the association between PPI use and hepatic steatosis, independent of alcohol consumption. The effect of PPIs on MASLD appears to be mediated mainly by increased lactic acid bacteria abundance, and is potentially, in part, serially mediated by endogenous ethanol production.

## Introduction

Proton pump inhibitors (PPIs) are one of the most commonly prescribed medications in the world. They are primarily used prophylactically against ulcers and for the treatment of gastroesophageal reflux disease.^1^ While they are generally regarded as safe, long-term use of PPIs has been marked for its potential adverse side effects.^2^ These side effects include various infections, nutrient deficiency, and small intestinal bacterial overgrowth.^3^ Another association for PPI use is an increased risk for and aggravation of liver diseases.^4-9^ While PPI use in humans is confounded by other risk factors, such as BMI, type 2 diabetes, and age, these factors do not fully explain the association between PPIs and steatotic liver disease (SLD). In mice, PPIs were shown to induce microvesicular steatosis after 60 d of treatment.^10^

The main subclassifications of SLD are metabolic dysfunction-associated steatotic liver disease (MASLD), alcohol-associated liver disease (ALD), and SLD with specific aetiology.^11^ MASLD is defined as the presence of hepatic steatosis in conjunction with one cardiometabolic risk factor and no other discernible cause. The association between PPI and SLD is independent of alcohol consumption and the other specific aetiologies and linked with the aetiology of MASLD and its corresponding cardiometabolic risk factors.^8^

It has been suggested that PPI-induced alterations in the gut microbiome might constitute a mediating factor. PPIs have been shown to be one of the strongest modulators of the gut microbiome,^7^^,^^8^^,^^12-15^ and changes in the microbiome have been linked to different stages in MASLD development.^8^^,^^16-18^ PPI-induced alterations of the microbiome are characterized by an increase in aerotolerant and facultative anaerobic microbes in the fecal matter. These microbes are predominantly from the bacterial orders Enterobacterales and Lactobacillales, which are commonly referred to as lactic acid bacteria. Bacteria from these orders have their primary ecological niche in the upper gastrointestinal tract, where substrate availability is high but extremely dynamic. Besides being increased with PPI use,^13^ these same taxonomic groups have also been linked to MASLD independent of PPI use.^16^ Both bacterial clades have adaptive metabolic capacities to respond to changing nutrient availability, which aligns with the dynamic setting of the small intestine, where substrate availability is extremely dynamic throughout the course of a day.

A well-known adaptive metabolic switch is between lactate fermentation and mixed acid fermentation. Lactate fermentation, which is less efficient in terms of ATP yield but supports faster growth, is preferential during high substrate availability. When growth is reduced due to depleting substrate availability or other environmental factors, such as low pH, mixed acid fermentation is preferred.^19^^,^^20^ Complete fermentation of sugars such as glucose and fructose through the mixed acid fermentation pathway results in the production of acetate, formate, and ethanol. Ethanol abuse, besides hepatic viral infections, has been one of the leading causes of steatotic liver disease. The potential of these microbes to produce clinically relevant levels of endogenous ethanol and concomitant association with MASLD was shown in various studies as well.^21-23^

These data indicate that the association between PPI and MASLD might, at least in part, be mediated by microbially derived endogenous ethanol. However, a thorough mediation analysis has not been performed. Here, we further investigated the potential mediatory effect of the microbiota between PPI use and MASLD and post-prandial plasma ethanol concentrations from our previous study.^23^ Furthermore, we perform a fecal microbiome mediation analysis between PPI use and the fatty liver index (FLI) in a general population cohort to test whether the association holds in a general population. The strongest associations were validated in the independent FINRISK cohort.

## Methods

### Study design and cohort characteristics

Data for this study were obtained from three different cohorts: the BARIA study,^24^ the HELIUS study,^25^ and the FINRISK 2002 study.^26^ The BARIA and HELIUS data served as discovery cohorts, and the FINRISK data were used for validation. The BARIA study included morbidly obese patients from the Netherlands who were scheduled for bariatric surgery, while the HELIUS and FINRISK studies involved population-based cohorts from the Amsterdam area and Finland, respectively. Data from the BARIA subjects were collected from 2016 until 2019. Data from the FINRISK 2002 study were obtained from samples obtained between January and March 2002. For HELIUS, baseline data were collected from 2011 to 2015, while follow-up data were collected between 2019 and 2022. Microbiome data and PPI use data from the HELIUS study were collected at baseline while outcome metrics were based on data collected during follow-up.^27^^,^^28^ Further detailed cohort information can be found in the corresponding manuscripts. Ethical approval was obtained from the Academic Medical Center Ethical Review Board for the BARIA (NL55755.018.15) and HELIUS (NL32251.018.10) studies. The FINRISK study was approved by the Coordinating Ethical Committee of the Helsinki and Uusimaa Hospital district (558/E3/2001). All participants provided written informed consent.

### Exposure and microbiome mediators

Anthropometric measurements, including height, weight, and waist and hip circumferences, were recorded during visits for physical examination. Fasting blood samples were collected to determine the levels of hemoglobin, HbA1c, glucose, triglycerides, alanine aminotransferase, aspartate aminotransferase, and gamma-glutamyl-transpeptidase. FLI scores were calculated using BMI (kg/m^2^), waist circumference (cm), triglycerides (mg/dL), and serum gamma-glutamyl transpeptidase (GGT) levels.^29^ Participants of the BARIA study underwent a 2-h mixed meal tolerance test (MMT), which consisted of two Nutridrink compact 125  mL (Nutricia®). An enzyme-based kit from DiaSys (Holzheim, Germany) was used to assay ethanol concentrations in blood. During bariatric surgery wedge liver biopsies were obtained. Liver histological sections were stained with hematoxylin-eosin and Sirius red and then reviewed by two experienced pathologist in tandem according to the steatosis, activity and fibrosis (SAF) score.^30^ Difficult borderline cases were discussed during panel meetings for consensus. PPI use in BARIA was retrieved from free text on medication use, registered by a physician on the day of the physical examination, and included the use of omeprazole, pantoprazole, and esomeprazol. HELIUS participants were asked to bring their prescribed medications, which were coded according to the Anatomical Therapeutic Chemical (ATC) classification (A02BC). In FINRISK, PPI was determined by (baseline) ATC code A02BC in the Social Insurance Institution of Finland (KELA) drug purchase prescription register. A02BC includes the use of the most common PPIs, omeprazole, pantoprazole, lansoprazole, rabeprazole, and esomeprazole. Fecal microbiome profiles were retrieved from previous efforts. For the BARIA study were obtained by metagenome sequencing,^23^ for the HELIUS study by 16S rRNA amplicon sequencing^31^ and for the FINRISK study by shallow metagenome sequencing.^32^ Alcohol consumption categories were assigned based on self-reported alcohol consumption. In HELIUS and BARIA, participants were categorized based on their sex and units of alcohol per week. For women, the criteria were, less than 3, between 3 and 7, or more than 7, while for men it was less than 5, between 5 and 11, or above 11 units of alcohol per week. In FINRISK, the criteria for women were less than 7 alcohol units, medium between 7 and 11, and high above 11. For men less than 14, between 14 and 22, 22 and above. Drinking more than 14 units per week was an exclusion criteria of the BARIA study. This is all in line with the SLD nomenclature, categorizing the participants into the MASLD classification.

### Outcomes

Biopsy-proven MASLD, the gold standard for MASLD diagnosis and classification, was only available for the bariatric cohort. Furthermore, we scrutinized the microbiome mediation potential between PPI use and postprandial ethanol levels in this cohort.^23^ The fatty liver index (FLI) score, which is reportedly the best non-invasive test to predict hepatic steatosis, was used in the general population cohorts.^33^ PPI use is known to aggravate the effects of ethanol consumption on liver disease; therefore, final mediation models were corrected for self-reported weekly consumption of alcoholic beverages to identify MASLD-associated microbiota.^4^

### Microbiome mediation analysis

Statistical analysis and visualizations were performed in R (4.3.2).^34^ For all cohorts, only participants for whom complete datasets where available were used. Metagenomic taxa abundances were scaled to relative abundance while 16S data from HELIUS was subsampled to 14,932 counts before scaling. Alpha diversity metrics were calculated using phyloseq (1.46.0)^35^ and beta diversity was tested using permutational multivariate analysis of variance from vegan (v.2.6.4)^36^ with Bray‒Curtis dissimilarity matrices. For univariate analysis, only taxonomies with at least 10% prevalence were tested. Linear models (stats 4.3.2) were used to evaluate the effects of PPI use on taxa abundance and the effects of taxa abundance on steatosis, post-prandial ethanol levels and FLI scores. Mediation analysis for all taxonomic ranks was performed using the mediation package (4.5.0)^37^ while the alcohol consumption-corrected validation models were evaluated using lavaan (0.6.19)^38^ and psych (2.3.12).^39^ To determine confidence intervals and *p*-values of the mediation models, a non-parametric bootstrap was used with 1000 simulations.

## Results

### Study design and cohort characteristics

**Figure 1.**
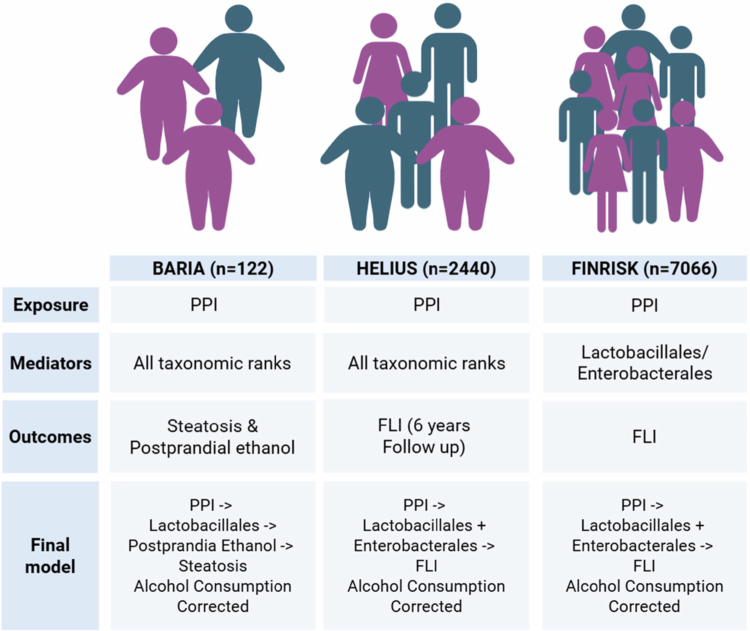
Diagram of the study design with three study cohorts: BARIA, HELIUS, and FINRISK, with their exposures, mediators, outcomes, and final model.

To determine the effect of the intestinal microbiota in the association between PPI use and MASLD, a mediation analysis was performed on two different cohorts consisting of bariatric surgery patients (*n* = 122) and a general population (HELIUS, *n* = 2440). The results from HELIUS were validated in an independent general population cohort (FINRISK, *n* = 7066) (Figure 1).

Participants from the BARIA study that did not have post-prandial ethanol measurements were excluded from the study. The descriptive characteristics of the cohorts were stratified by PPI use and cardiometabolic and liver metrics, and potential confounders were assessed (Table 1).

**Table 1.** Cohort characteristics by PPI use. Values represent mean (± SD) or frequency (% within group), statistical testing was done using t-test for continuous variables or chi-square test for categorical variables.

		BARIA (*N* = 122)			HELIUS (*N* = 2440)			FINRISK (7066)		
		No PPI	PPI		No PPI	PPI		No PPI	PPI	
Characteristic	units	(*N* = 87)	(*N* = 35)	*p*-value	(*N* = 2193)	(*N* = 247)	*p*-value	(*N* = 6872)	(*N* = 194)	*p*-value
Age	Years	45 (± 9.4)	50 (± 10)	**0.029**	51.0 (± 10.6)	55.9 (± 8.1)	**<0.001**	49.2 (± 13.0)	55.9 (± 11.1)	**<0.001**
Sex	Male	16 (21%)	9 (29%)	0.504	1056 (48%)	110 (45%)	0.290	3767 (55%)	118 (60%)	0.113
	Female	61 (79%)	22 (71%)		1123 (52%)	136 (55%)		3105 (45%)	76 (40%)	
BMI	kg/m2	40 (± 4.0)	39 (± 3.7)	0.218	27.1 (± 4.6)	28.8 (± 5.0)	**<0.001**	27.0 (±4.7)	28.6 (±5.1)	**<0.001**
Waist	cm	120 (± 12)	120 (± 10)	0.8	92.9 (± 12.2)	97.6 (± 12.8)	**<0.001**	89.4 (±13.4)	93.8 (±14.4)	**<0.001**
Triglycerides	mmol/L	1.6 (± 1.0)	1.7 (± 0.98)	0.39	1.1 (± 0.7)	1.3 (± 0.9)	**0.003**	1.4 (±0.9)	1.7 (± 1.6)	**0.01**
LDL	mmol/L	3.2 (± 0.89)	2.7 (± 1.1)	**0.027**	3.2 (± 0.9)	3.0 (± 1.0)	**0.002**	3.4 (± 0.9)	3.4 (± 0.9)	0.583
GGT	U/L	28 (± 15)	54 (± 57)	**0.018**	29.1 (± 27.0)	34.8 (± 42.9)	**0.042**	33.3 (± 36.0)	38.1 (± 48.7)	0.178
ASAT	U/L	25 (± 8.1)	31 (± 13)	**0.014**	26.1 (± 9.3)	26.1 (± 7.7)	0.979	27.6 (± 24.9)	28.7(± 21.0)	0.54
ALAT	U/L	31 (± 16)	44 (± 28)	**0.019**	25.2 (± 14.6)	25.0 (± 11.9)	0.775	26.0 (± 18.6)	25.6 (± 14.1)	0.670
FLI score	[-]	94 (± 5.8)	95 (± 5.4)	0.377	39.8 (± 27.6)	51.5 (± 29.8)	**<0.001**	41.8 (±30.1)	52.4 (±31.2)	**<0.001**
HbA1c	mmol/mol	41 (± 9.8)	44 (± 10)	0.171	39.3 (± 7.8)	42.5 (± 8.8)	**<0.001**	37.1 (± 7.0)	38.6 (±7.1)	**0.027**
Glucose	mmol/L	6.0 (± 1.6)	6.7 (± 1.8)	0.082	5.5 (± 1.0)	5.8 (± 1.3)	**0.001**	5.9 (± 1.1)	6.1 (± 1.4)	0.26
Diabetes	Yes	10 (13%)	12 (39%)	**0.006**	171 (8%)	53 (22%)	**<0.001**	249 (3.6%)	12 (6.2%)	0.094
	No	67 (87%)	19 (61%)		1996 (92%)	193 (78%)		6623 (96%)	182 (94%)	
Cardiometabolic Risk Factors	0/1/2/3/4/5 (%)	0/0/23/40/29/9	0/0/7/51/33/9	0.097	14/23/27/25/8/2	4/14/23/35/18/6	**0.003**			
Alcohol use^#^	Low	80 (92%)	32 (91%)	0.78	1513 (69%)	187 (76%)	0.065	3652 (55%)	115 (64%)	0.059
	Moderate	6 (7%)	2 (6%)		485 (22%)	44 (18%)		1646 (25%)	35 (20%)	
	High	1 (1%)	1 (3%)		175 (8%)	13 (5%)		1305 (20%)	29 (16%)	
Steatosis	Yes	50 (57%)	25 (71%)	0.22						
	No	37 (43%)	10 (29%)							
Ethanol Fasted	µmol/L	47.7 (±24.8)	58.7 (±23.0)	**0.022**						
Ethanol post-prandial	µmol/L	76.9 (±45.3)	106.7 (±51.9)	**<0.01**						

Note: The alcohol consumption categories were low, moderate, and high (units/week): BARIA & HELIUS – women <3, 3–7, >7; men <5, 5–14, >14; FINRISK – women <7, 7–11, >11; men <14, 14–22, >22; alcohol intake >14 units/week was an exclusion criterion in BARIA.

### Lactobacillales mediate PPI-associated with post-prandial ethanol levels and steatosis

The first cohort consisted of 122 obese bariatric surgery patients, 35 of whom were using PPIs. The subjects who used PPIs were older, had lower LDL levels, had elevated levels of GGT, ASAT and ALAT, and were also more likely to have diabetes mellitus type 2. While relatively more PPI users had steatosis, this difference was not significant (Table 1; *p* = 0.22), and only the genus of *Limosilactobacillus* showed significant mediation effect (Supplementary Figure 1).

**Figure 2.**
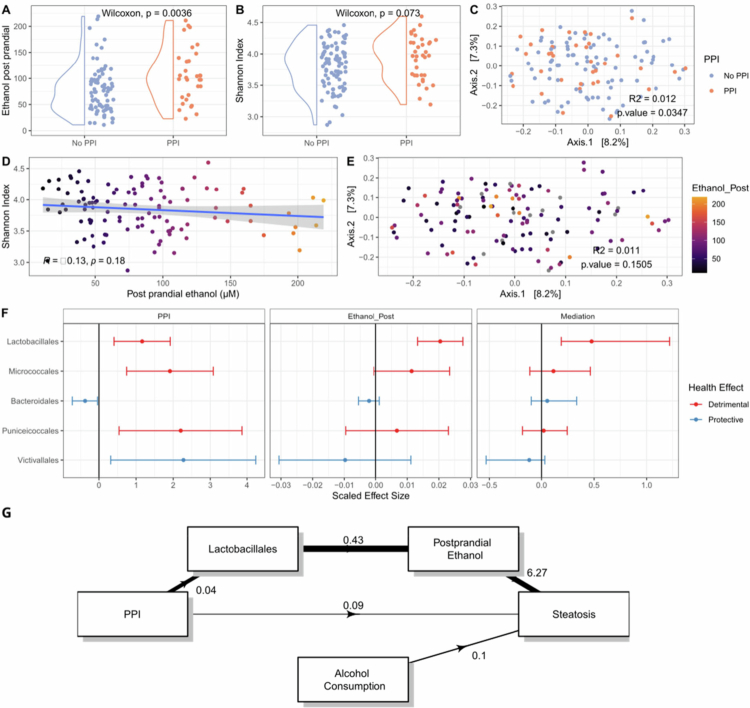
Results from the mediation PPI microbiome analysis of the BARIA cohort. (A) Difference in post-prandial ethanol concentrations between No PPI and PPI users. (B) Microbiome alpha diversity. (C) Beta diversity represented by PCoA of the Bray‒Curtis dissimilarity. Associations between post-prandial ethanol concentrations and (D) alpha diversity and (E) beta diversity represented by a PCoA of the Bray‒Curtis dissimilarity. (F) Scaled effect sizes of PPI use on the relative abundance of gut microbial orders. The effect sizes of these bacterial orders on post-prandial ethanol concentrations. And the mediation effect of these bacterial orders on the effects of PPIs on post-prandial ethanol concentrations. Only orders with significant associations with ethanol or PPIs are shown. (G) Mediation diagram, the width of the arrow indicates the standardized effect size.

However, ethanol levels were increased in fasted (*p* = 0.022) and especially post-prandial (*p* < 0.01) plasma samples of PPI users (Figure 2A). PPI use did not significantly associated with microbiome alpha diversity (Shannon index, *p* = 0.073, Figure 2B) but did with composition (Bray‒Curtis dissimilarity, R2 = 0.012 *p* = 0.039, Figure 2C) and various taxa (Figure 2F, Supplementary Table 1). Endogenous ethanol is a likely metabolic mediator between the microbiome and MASLD and a serial mediator in the association between PPI use and MASLD. As previously reported, post-prandial ethanol did not associated with microbiome alpha-diversity (Shannon index, *p* = 0.18, Figure 2D) and composition (Bray‒Curtis dissimilarity, R2 = 0.011, *p* = 0.1458, Figure 2E), but various taxa did show an association (Figure 2F, Supplementary Figure 2). Especially, Lactobacillales showed a high mediation potential (proportion mediated = 0.479; *p* = 0.008; Figure 2F). While fungi have been implicated in auto-brewery syndrome, no significant associations with PPI use (*p* = 0.45), post-prandial ethanol concentrations (*p* = 0.70) or MASLD (*p* = 0.56) status were found.

Taken together, we constructed one final model in which we included a serial mediated path between PPI use and steatosis grade through Lactobacillales and post-prandial ethanol. While no direct effect of PPI use on Steatosis was found (*p* = 0.62), serial mediation analysis showed that the mediation effect of PPI through Lactobacillales abundance and post-prandial ethanol on steatosis was significant (*p* = 0.007) (Figure 2G).

### Aerotolerant microbes mediate the effects of PPI on FLI

Since the small bariatric surgery cohort consisted of patients with severe obesity, the results might not generalize to the general population. Therefore, we further explored the associations between PPI use, the fecal microbiome, and hepatic steatosis in a general multi-ethnic population cohort. From the Healthy Life in an Urban Setting (HELIUS) cohort,^25^ data were obtained for 2440 participants for which both microbiome data at baseline and FLI scores at follow-up were available. A total of 247 participants were using PPIs at baseline, coinciding with the time of fecal sample collection

**Figure 3.**
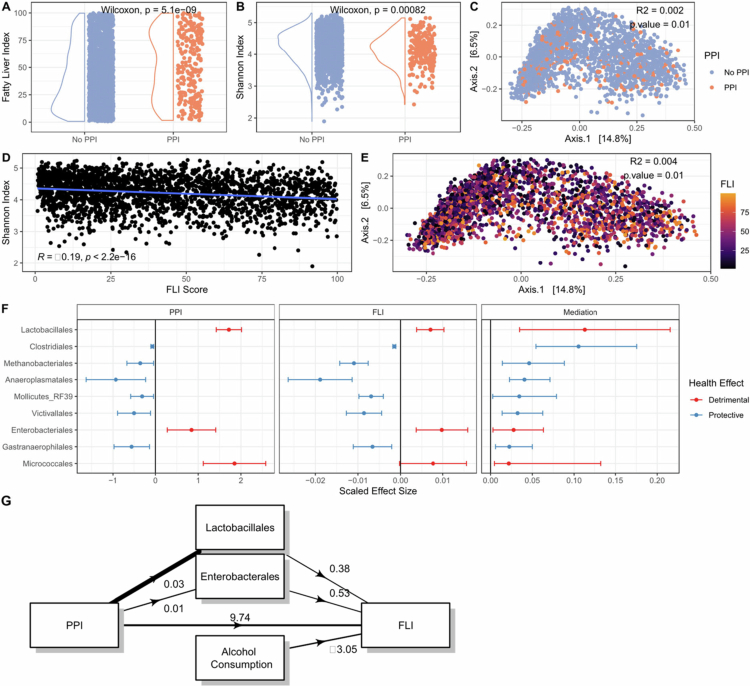
Associations between PPI use, FLI, and the microbiome in the HELIUS study. (A) FLI score distributions stratified by PPI use. (B) Shannon index distributions stratified by PPI use. (C) PCoA of the Bray‒Curtis dissimilarity. (D) Scatter plot showing the negative relation between FLI score and Shannon index. (E) PCoA of the Bray‒Curtis dissimilarity and its association with the FLI score. (F) Relative effects of PPI use and FLI score on the microbial abundance of different bacterial orders with significant mediation potential. Lines indicate 95% confidence intervals. (G) Mediation diagram, the width of the arrow indicates the standardized effect size.

Similar to the bariatric surgery cohort, participants using PPI were older, had lower LDL levels and had higher triglycerides, GGT, and glucose values. They also had higher fasting glucose and HbA1c levels and were more likely to be diabetic. FLI scores were significantly (*p* < 0.001) higher in the PPI use group (Figure 3A). Microbiome alpha-diversity was significantly reduced (Shannon index, *p* < 0.001, Figure 3B), and the composition was altered with PPI use (Bray‒Curtis dissimilarity, R2 = 0.002, *p* = 0.01, Figure 3C). Similarly, microbiome alpha-diversity was reduced (Pearson Rho = -0.19; *p* < 0.001; Figure 3D), and composition was associated with increased FLI scores (Bray‒Curtis dissimilarity, R2 = 0.004; *p* = 0.01; Figure 3E). PPI use was associated with an increase in oxygen-tolerant microbes that thrive in the small intestine, and decreased strict anaerobes (Figure 3F, Supplementary Table 2). These same taxa were associated with increased FLI scores. The microbial order with the highest mediation estimate was Lactobacillales (proportion mediated = 0.11; *p* = 0.004). Methanobacteriales, Clostridiales, Victivallales Anaeroplasmatales, and Enterobacterales also had significant mediating effects (Figure 3F, Supplementary Figure 3). A summary mediation model in which we included both Lactobacillales and Enterobacterales and corrected for ethanol consumption was constructed (Figure 3G). This model showed that only Lactobacillales mediated the association (+1.04, *p* = 0.02) and not Enterobacterales (+0.28, *p* = 0.096), while alcohol consumption had a negative effect on FLI scores (−3.05, *p* = 0.001).

### Validation of Lactobacillales mediation

Though the mediation effect of the order of Lactobacillales was only nominally significant, it shows the strongest mediation potential in both datasets. The HELIUS results suggest that species from the order of Enterobacterales may also act as a significant mediators. Therefore, the mediation effect of only these two orders were validated in the FINRISK dataset using a single mediation model. Additionally, the effects of alcohol consumption were included to correct for the potential confounding mediation effect.

In this Finnish cohort, fecal microbiome data and FLI scores were available for 7066 subjects of which 194 were reported to take PPIs. Compared to HELIUS the relative use of PPI was four times lower, reflecting still moderate use in 2002.^40^ Similar to the HELIUS cohort subjects on PPIs in this cohort were older, had higher BMI and waist circumference than those not on PPIs. They furthermore showed higher levels of triglycerides and HbA1c levels, though diabetes prevalence did not differ.

**Figure 4.**
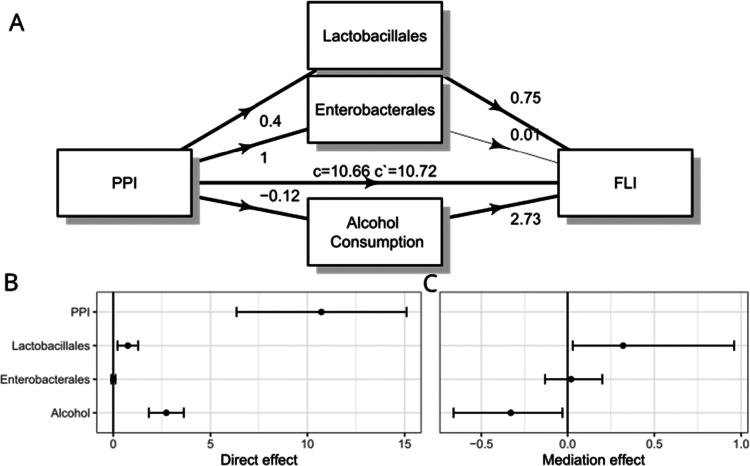
Validation of the mediation analysis between proton pump inhibitor (PPI) use and the Fatty Liver Index (FLI) in the FINRISK cohort. A mediation diagram, significant associations are indicated with bold arrows. (B) Direct effects of exposure and mediators on FLI, mean, and 95% confidence intervals. (C) Mediation effect with boot-strapped value and lower and upper limits.

The mediation analysis showed again that PPI use was associated with a 10-point increase in the FLI score (10.66, *p* = 10^−6^) and increases in the prevalence of fecal Lactobacillales (+0.4%, *p* = 7.7 × 10^−4^) and Enterobacterales (+1.0%, *p* = 6.8 × 10^−3^) (Figure 4A). Alcohol consumption was negatively associated with PPI use and showed a negative mediation effect, indicating that the effects of PPI use are in part countered by reduced exogenous ethanol consumption. Lactobacillales had a significant direct effect on the FLI score (+0.75, *p* = 0.005) and significantly mediated the association between PPI and FLI (+0.32, *p* = 0.005) while Enterobacterales did not show a significant direct or mediation effect on FLI scores (+0.01, *p* = 0.85) (Figure 4B).

## Discussion

There is an increased risk for MASLD with PPI use, which is only in part explained by BMI and other confounding factors.^6^^,^^8^ Microbes enriched after PPI use are also associated with MASLD, indicating a potential microbiota-mediated effect. The PPI-enriched microbial lineages are usually oxygen tolerant or facultative anaerobes from the upper GI tract, mainly from the orders of Lactobacillales and Enterobacterales.^13^ The results from this study indicate that especially lineages from the order of Lactobacillales mediate the association between PPI use and MASLD. While mainly known for their lactate production, various strains of this order are known to produce ethanol as a fermentation product. Taken together, these results indicate a serially mediated path between PPI use and MASLD through Lactobacillus-derived endogenous ethanol.

### Altered small intestinal ecological niche

The targets of PPIs and the differences in fecal microbiome composition indicate that the microbial ecology of the stomach and small intestine is most affected by PPI use. With increased gastric pH, microbes are more likely to survive passage through the stomach or are even able to replicate in this environment. With more bacterial biomass at the beginning of the small intestine, reduced pH shock after gastric emptying and high nutrient availability, increased proliferation of small intestinal microbes is to be expected with the use of PPIs. This is substantiated by the fact that PPI use is also implicated in the development of small intestinal bacterial overgrowth (SIBO).^41^^,^^42^ The hallmark microbes of SIBO include Klebsiella, Escherichia and Enterococcus while SIBO itself also increases the risk of MASLD.^18^^,^^43^ While the pH in the stomach is significantly increased after PPI use, the distal small intestinal pH is reduced^44^ and likely reflects increased microbial fermentation activity. Additionally, gastric motility disorders in patients with type 2 diabetes are strongly associated with MASLD.^45^ The rapid increase in plasma ethanol concentrations within two hours after a meal furthermore implicates the small intestine as the main site for ethanol production.^23^ Therefore, increased microbial activity in the small intestine is a likely driver in PPI-associated MASLD development.

### Intestinal ethanol fermentation

A characteristic of the microbiota of the small intestine is their capacity for rapid conversion of simple carbohydrates through lactate and mixed acid fermentation.^46^ Lactate fermentation is fast and results in a net production of two ATP molecules per glucose consumed. Mixed acid fermentation, in which ethanol is one of the major end products, results in three ATP produced though at a much lower rate. Switching between these pathways is a common strategy to address the fast and feast dynamic setting of the small intestine. This means that when substrate availability is high, lactate fermentation is preferred. When growth rates are reduced due to limiting substrate availability or other environmental factors, mixed acid is preferred.^19^^,^^47^ These findings suggest that the distal small intestine or proximal colon are the main sites of intestinal ethanol production.

### Ethanol-producing lineages

Strains from the orders Enterobacterales and Lactobacillales have the capacity to adapt their metabolism, produce ethanol, and thrive in the small intestine.^47^ However, we found that mainly Lactobacillales mediate the effects of PPI on endogenous ethanol production and MASLD or SLD. Another European study also found an association with ethanol production and *Lactobacillus fermentum,*^22^^,^^23^ while a US-based study found *Enterococcus faecalis* to produce ethanol and aggravate alcohol-related liver disease.^4^ In contrast, ethanol derived from *Klebsiella pneumoniae,^21^* a member of the Enterobacterales, is implicated in MASLD development in Asian studies. These findings indicate that other factors, in addition to PPI use, that differ geographically and culturally, act upon the small intestinal ecosystem, might drive endogenous ethanol production.

### Clinical implications

While PPI use is generally regarded as safe, there are negative side effects with long-term use, such as nutrient malabsorption and MASLD.^2^ Therefore, inappropriate continuation, which is highly prevalent for PPI use,^48^ should be guarded against. Alternatively, H2-receptor agonists should be considered, which have a less pronounced effect on the microbiome and are associated with a lower prevalence of MASLD.^49^^,^^50^ Complementing the use of PPIs with probiotic strains that prefer the same ecological niche, but without ethanol production capacity, might be a viable strategy to counter the proliferation of ethanol-producing strains.^51^ Additionally, dietary interventions strategies might be used to control the fermentation activity of the microbiota. While the focus of this study was on MASLD, these microbiome mediating effects might also explain the aggravating effects of PPI use in ALD.

### Limitations

The major limitation of the study is the use of FLI scores in the general population cohorts as a proxy for MASLD. The FLI is a composite score based on BMI, waist circumference, and GGT and triglyceride levels. BMI is a major confounder for both MASLD and PPI use, but could not be corrected for with the current study design. Furthermore, potential residual confounding of other factors cannot be excluded. Secondly, only in the bariatric surgery cohort post-prandial ethanol levels were available and we were not able to validate this serial mediation effect in the general population cohorts. Future studies could validate the association between PPI use and endogenous ethanol using intervention studies or measure the urinary marker phosphatidyl ethanol (PEth) for ethanol exposure, whether endogenous or exogenous. While the results indicate that small intestinal ecology is primarily affected by PPI use, only fecal samples were available for microbiome profiling. The data used came from European cohorts, and though the HELIUS is a multi-ethnic cohort, the results might not generalize to other geographic regions. In the HELIUS cohort, alcohol consumption was negatively associated with FLI scores, which can be attributed in part to the ethnic make-up.^52^

## Conclusion

Lactobacillales showed the strongest mediating potential of the gut microbiome in the association between PPI use and MASLD. The association could, in part, be serially mediated by endogenous ethanol production.

## Supplementary Material

Supplementary_Figures.docx Supplementary_Figures.docx

Supplementary_table_2.xlsx Supplementary_table_2.xlsx

Supplementary_table_1.xlsx Supplementary_table_1.xlsx

## Data Availability

The HELIUS data are owned by the Amsterdam University Medical Centers, which are located AMC in Amsterdam, The Netherlands. Any researcher can request the data by submitting a proposal to the HELIUS Executive Board as outlined at http://www.heliusstudy.nl/en/researchers/collaboration, by email: heliuscoordinator@amsterdamumc.nl. The HELIUS Executive Board will check proposals for compatibility with the general objectives, ethical approvals and informed consent forms of the HELIUS study. There are no other restrictions to obtain the data, and all data requests will be processed in the same manner.
