# Supplementary material for: Lactic acid bacteria and endogenous ethanol mediate proton pump inhibitor-associated MASLD: a multicohort cross-sectional mediation analysis
Source: Gut Microbes. 2026 May 3;18(1):2664712. doi: 10.1080/19490976.2026.2664712 (PMC13154942; doi:10.1080/19490976.2026.2664712)
Supplement: Supplementary_Figures.docx [file KGMI_A_2664712_SM4338.docx]

# Supplementary Figures


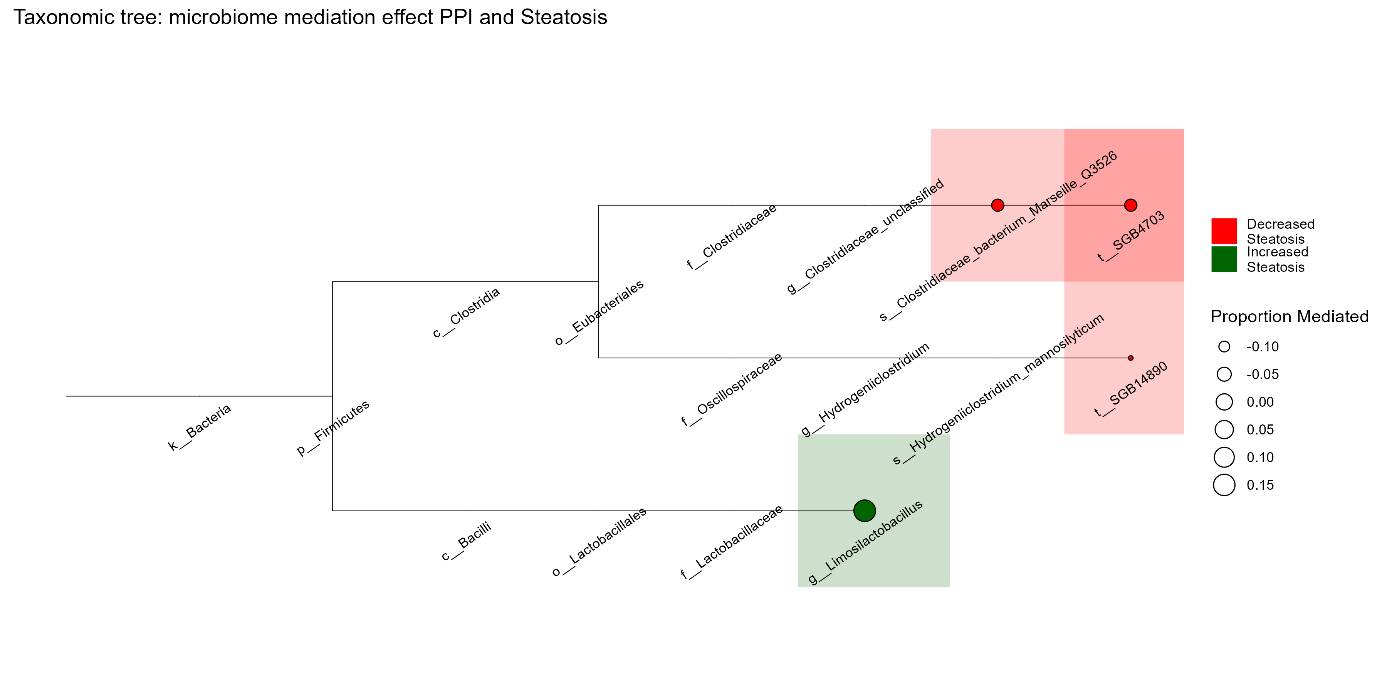
Figure S1; Taxonomic tree highlighting taxa that mediate the association between PPI use and steatosis in the BARIA cohort. Only tips and nodes for which the association was significant are shown. Clades in red are reduced with PPI use and have a negative effect on steatosis grade while clades in green are increased with PPI use and show a positive effect on steatosis grade.


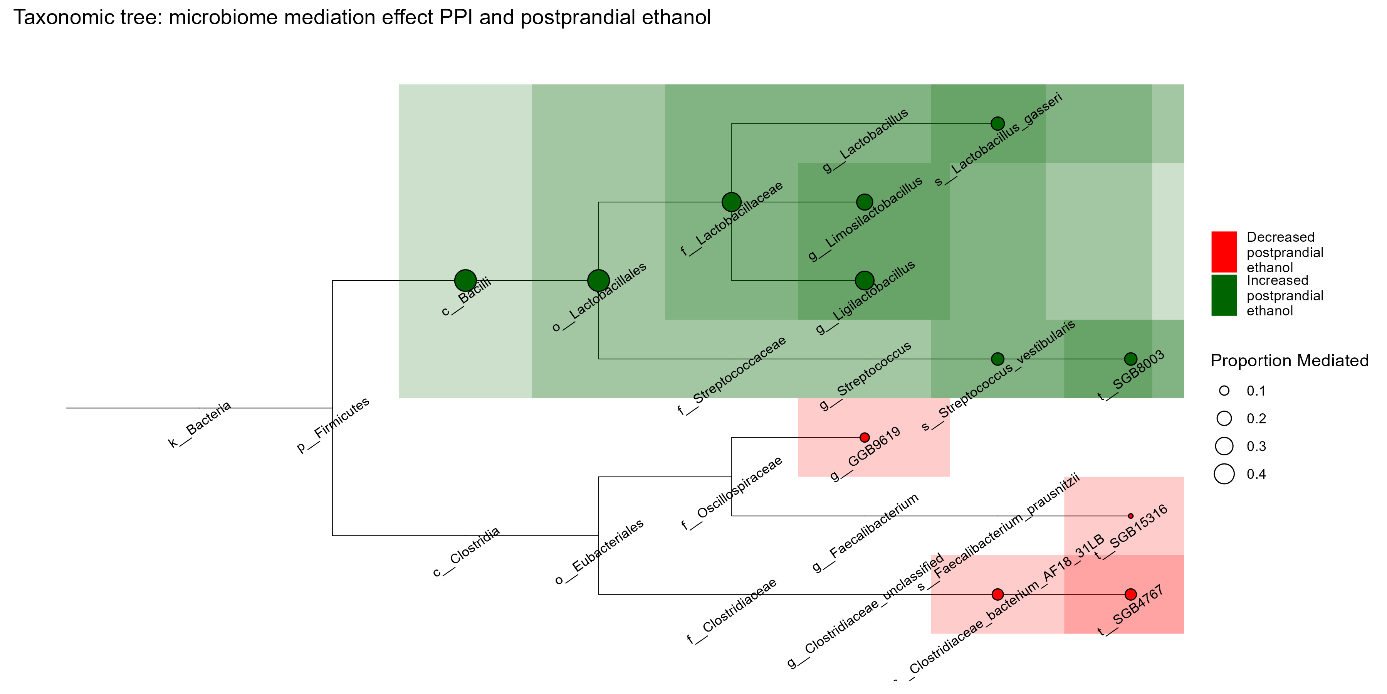


Figure S2. Taxonomic tree highlighting taxa that mediate the association between PPI use and postprandial ethanol in the BARIA cohort. Only tips and nodes for which the association was significant are shown. Clades in red are reduced with PPI use and have a negative effect on post-prandial ethanol levels, while clades in green are increased with PPI use and show a positive effect on postprandial ethanol.


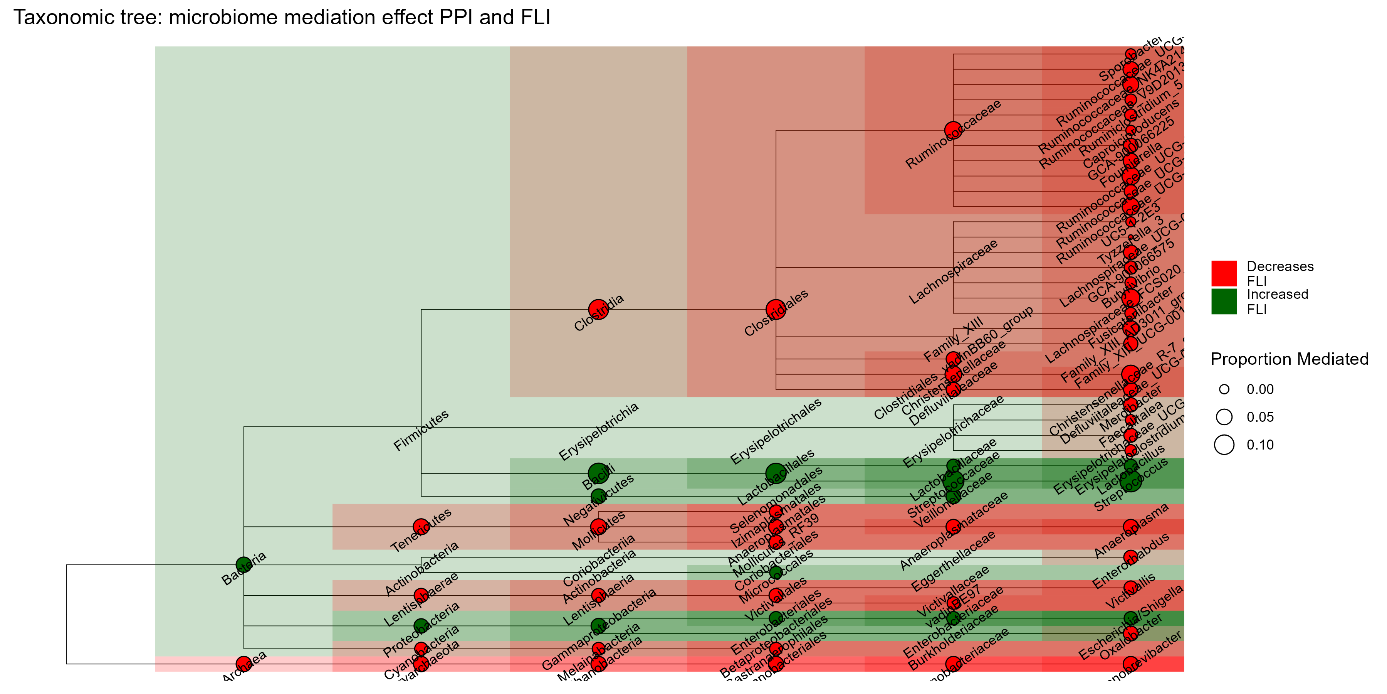


Figure S3. Taxonomic tree highlighting taxa that mediate the association between PPI use and FLI in the HELIUS cohort. Only tips and nodes for which the association was significant are shown. Clades in red are reduced with PPI use and have a negative effect on post-prandial ethanol levels, while clades in green are increased with PPI use and show a positive effect on postprandial ethanol.
